# DeepCarc: Deep Learning-Powered Carcinogenicity Prediction Using Model-Level Representation

**DOI:** 10.3389/frai.2021.757780

**Published:** 2021-11-18

**Authors:** Ting Li, Weida Tong, Ruth Roberts, Zhichao Liu, Shraddha Thakkar

**Affiliations:** ^1^ Division of Bioinformatics and Biostatistics, National Center for Toxicological Research, US Food and Drug Administration, Jefferson, AR, United States; ^2^ University of Arkansas at Little Rock and University of Arkansas for Medical Sciences Joint Bioinformatics Program, Little Rock, AR, United States; ^3^ ApconiX Ltd., Alderley Edge, United Kingdom; ^4^ Department of Biosciences, University of Birmingham, Birmingham, United Kingdom; ^5^ Office of Translational Sciences, Center for Drug Evaluation and Research, US Food and Drug Administration, Silver Spring, MD, United States

**Keywords:** carcinogenicity, deep learning, QSAR, non-animal models, NCTRlcdb

## Abstract

Carcinogenicity testing plays an essential role in identifying carcinogens in environmental chemistry and drug development. However, it is a time-consuming and label-intensive process to evaluate the carcinogenic potency with conventional 2-years rodent animal studies. Thus, there is an urgent need for alternative approaches to providing reliable and robust assessments on carcinogenicity. In this study, we proposed a DeepCarc model to predict carcinogenicity for small molecules using deep learning-based model-level representations. The DeepCarc Model was developed using a data set of 692 compounds and evaluated on a test set containing 171 compounds in the National Center for Toxicological Research liver cancer database (NCTRlcdb). As a result, the proposed DeepCarc model yielded a Matthews correlation coefficient (MCC) of 0.432 for the test set, outperforming four advanced deep learning (DL) powered quantitative structure-activity relationship (QSAR) models with an average improvement rate of 37%. Furthermore, the DeepCarc model was also employed to screen the carcinogenicity potential of the compounds from both DrugBank and Tox21. Altogether, the proposed DeepCarc model could serve as an early detection tool (https://github.com/TingLi2016/DeepCarc) for carcinogenicity assessment.

## Introduction

It is crucial to assess the carcinogenic potency for chemicals, an important factor that triggers regulatory actions for both new and existing chemicals. In 1995, the ICH′ Guideline on the Need for Carcinogenicity studies of Pharmaceuticals was introduced and outlined the need, study design, and interpretation for carcinogenicity studies. Essentially, since carcinogenicity studies are time-consuming and resource-intensive, they should only be performed when human exposure warrants the need for information from lifetime studies in animals to assess carcinogenic potential (ICHS1A, 1995) (Guideline, 1996). Generally, the experimental approach requires a long-term carcinogenicity study (104 weeks) in the rodent plus one other study that supplements the main study (ICHS1B, 1997) (Guideline, 1998), which can be a second-long term study or a shorter study (29 weeks) in a second species. This more concise study could use a transgenic mouse bioassay or a model based on initiation-promotion (ICHS1B, 1997) (Guideline, 1998).

Irrespective of the choices around carcinogenicity studies, each of these studies, on average, requires ∼500 rodents and costs around $1.1 m. Moreover, there is evidence of flawed extrapolation for carcinogenicity. There have been many endeavors to address this issue, such as developing biomarkers for use in shorter-term studies as predictors of outcome (Yamamoto et al., 1998; Venkatachalam et al., 2001; Morton et al., 2002). However, these approaches still rely heavily on experimental animals and do not address the 3Rs (replacement, reduction, and refinement of animals in toxicology testing). Programs such as Horizon 2020, The Seventh Framework Programme 7 (FP7), Tox21, Horizon 2020 Precision Toxicology, and other public-private partnerships (Vinken et al., 2021) have offered innovative thinking on developing animal-free methodologies and offer improved translation to humans. These new approach methodologies combine in silico and *in vitro* approaches such as read-across (Shah et al., 2016), toxicogenomics (Yauk et al., 2020), and adverse outcome pathways (AOPs) (Yang et al., 2020).

Several studies have investigated the prediction of carcinogenic potency (Lee et al., 2003; Morales et al., 2006; Tanabe et al., 2010; Caiment et al., 2014; Toropova and Toropov, 2018). The use of the quantitative structure-activity relationship (QSAR) model has become increasingly important for risk assessment because it can provide a fast and economic evaluation of the toxicity of a molecule using only the chemical structure. Some of the QSAR models were developed for carcinogenicity assessment for particular chemical classes (i.e., aromatic amines, food-relevant phytochemicals, polycyclic aromatic hydrocarbon) (Franke et al., 2001; Benigni and Passerini, 2002; Franke et al., 2010; Glück et al., 2018; Li et al., 2019). Although the predictions of these models can vary with interpretation, the application of these models was limited to specific domains. Models for non-congeneric chemicals include various classes of chemicals, which are of great interest for regulatory use (Fjodorova et al., 2010; Zhang et al., 2016a; Zhang et al., 2017; Wang et al., 2020). For example, Zhang et al. (2016b) built a naïve Bayes classifier on 1,042 compounds with rat carcinogenicity and yielded an overall accuracy of 0.90 ± 0.008 and 0.68 ± 0.019 for the training set and external test set, respectively. Zhang et al. (2017) developed an ensemble XGBoost model using 1,003 compounds with rat carcinogenicity and reported an accuracy of 0.7, sensitivity of 0.65, and specificity of 0.77 in external validation. Wang et al. (2020) constructed a novel sparse data deep learning (DL) tool based on the 1003 compounds from Zhang’s study (Zhang et al., 2017) and yielded an accuracy of 0.85, sensitivity of 0.82, and specificity of 0.88. These models covered a wide range of chemical classes. However, the annotation of carcinogenicity was only based on the rat in these studies. Since the animal carcinogenicity assessment was required to be conducted at least on two rodent species, it would give a more robust annotation by combining the carcinogenicity signal from both rats and mice. Therefore, we used the National Center for Toxicological Research liver cancer database (NCTRlcdb) (Young et al., 2004), which compressed the carcinogenicity information from both genders of rats and mice.

Deep learning (DL) has been successfully applied to predict complex endpoints, such as drug-induced liver injury (DILI) (Hwang et al., 2020; Li et al., 2020; Semenova et al., 2020) and cardiovascular toxicity (Wang et al., 2017; Maher et al., 2020; Rashed-Al-Mahfuz et al., 2021; Zeleznik et al., 2021). We proposed the DeepDILI model to incorporate model-level representations produced by five different machine learning algorithms into a neural network framework for DILI prediction (Li et al., 2021). The proposed DeepDILI outperformed the publicly available chemical-based DILI prediction models developed from different machine learning (ML) algorithms. However, the DeepDILI study only applied one arbitrary strategy for base classifier selection. The more sophisticated and automatic base classifier selection strategies that should be implemented may further improve the DeepDILI model architecture for other toxicity assessments.

In this paper, we proposed a DeepCarc model to predict carcinogenicity for small molecules using DL based model-level representations. The carcinogenicity annotation was obtained from the NCTRlcdb, incorporating the carcinogenicity signals from both rats and mice. In addition to the previous arbitrary base classifier selection strategy, we also explored a new strategy to select robust base classifiers based on the training set and development set performance. The developed DeepCarc model was comprehensively compared with the optimized 5 ML classifiers, two state-of-the-art ensemble classifiers, and four DL models. In addition, we also employed the DeepCarc model in prioritizing chemicals for carcinogenic potency in the DrugBank and Tox21 chemical databases.

## Materials and Methods

### Data Preparation

To curate a list of compounds for DeepCarc model development, we employed the NCTRlcdb with liver-specific carcinogenicity (Young et al., 2004). The NCTRlcdb provided a single carcinogenicity call per compound, summarizing multiple records representing each gender, species, route of administration, and organ-specific toxicity from the Carcinogenic Potency Database (CPDB) (Gold et al., 1999). Additionally, NCTRlcdb removed inorganic compounds, mixtures, and organometallics from the CPDB to facilitate QSAR model development. In total, NCTRlcdb contained 999 compounds with seven carcinogenicity categories. We excluded compounds from four categories without clear carcinogenicity information, including associated, probable, equivocal, and no opinion. We only employed the compounds from the other three categories, including cancer-liver, cancer-other and negative. The compounds from cancer liver and cancer-other were considered as carcinogens, while compounds from negative were classified as non-carcinogens. More specifically, the non-carcinogens were the compounds without carcinogenic potency observed during reasonably thorough, chronic long-term tests (Gold et al., 1991). Duplicate compounds were removed by comparing their InChI keys. The final data set consisted of 863 compounds, of which 561 were carcinogens and 302 were non-carcinogens (Supplementary Table S1).

To assign the chemical structures uniformly and avoid potential data bias, we applied the Kennard-Stone (KS) (Kennard and Stone, 1969) algorithm to split the whole data set (i.e., 863 compounds) into the training set, development set, and test set. Consequently, the training set included 554 compounds (360 carcinogens/194 non-carcinogens), the development set contained 138 compounds (90 carcinogens/48 non-carcinogens), and the test set consisted of 171 compounds (111 carcinogens/60 non-carcinogens). The structure description file (SDF) of compounds was downloaded from PubChem (https://pubchem.ncbi.nlm.nih.gov/pc_fetch/pc_fetch.cgi) for molecular descriptor calculation (Kim et al., 2021).

### Chemical Representation

Three different types of descriptors were calculated for each compound: Mol2vec (Jaeger et al., 2018), Mold2 (Hong et al., 2008), and Molecular ACCess System (MACCS) (Durant et al., 2002) structural keys.

Mol2vec is an unsupervised ML approach trained on a corpus containing 19.9 million compounds to learn vector representations of molecular substructures (Jaeger et al., 2018). For chemical-related substructures, their vector representations point to similar directions in the high dimensional space. Compounds can be represented as vectors that add up from the vectors of the individual substructures. 300-dimensional vector representations were constructed for all compounds.

Mold2 (https://www.fda.gov/science-research/bioinformatics-tools/mold2) is a publicly available software for calculating 777 chemical-physical based 1D/2D descriptors from chemical structure (Hong et al., 2008). The Mold2 software enables a rapid calculation of these large and diverse descriptors. Compared with commercial software packages (Hong et al., 2008), it requires low computing resources to generate the Mold2 descriptors, which contain a similar amount of information.

MACCS is a substructure of keys-based fingerprints encoded as SMART patterns (Durant et al., 2002). Two versions are available, one with 960 structural keys and the other with 166 structure keys. The shorter one is more popular as it can be calculated by several software packages and includes most of the chemical features for drug discovery and virtual screening. A single binary bit value of the bit string indicates the presence or absence of a substructure in the compound.

Two steps of descriptor preprocessing were applied to these three chemical representations. First, we removed the descriptors with zero variance. Secondly, we only kept one descriptor if two descriptors had a pairwise correlation coefficient of more than 0.9. Consequently, 297 of 300 Mol2vec descriptors, 330 of 777 Mold2 descriptors, and 138 of 166 MACCS descriptors were kept for model development (Supplementary Table S2).

### Discrimination Ability of Chemical Representations

To investigate whether the three chemical representations have a discrimination ability to distinguish between carcinogens and non-carcinogens, we calculated the pairwise compound similarity within carcinogens and non-carcinogens in training and development sets, respectively. We applied the Tanimoto coefficient to calculate the degree of similarity of any two compounds, as it is an appropriate choice for similarity calculation (Willett, 2006; Bajusz et al., 2015). All three chemical representations, Mol2vec, Mold2, and MACCS, were used to calculate the similarity. The Tanimoto coefficient S_A_,_B_ of molecules A and B is calculated by Eq. 1 for the continuous variables (e.g., Mol2vec and Mold2) and Eq. 2 for dichotomous variables (e.g., MACCS).

$$S_{A,B}=\frac{\sum_{j=1}^{n}X_{jA}X_{jB}}{\sum_{j=1}^{n}{\left(X_{jA}\right)}^{2}\;+\;\sum_{j=1}^{n}{\left(X_{jB}\right)}^{2}-\sum_{j=1}^{n}X_{jA}X_{jB}}$$ (1)

$$S_{A,B}=\frac{c}{a+b-c}$$ (2)

Where $X_{jA}$ is the value of the j th feature in molecule A, $X_{jB}$ is the value of the j th feature in molecule B, a is the number of bits with value 1 in molecule A, b is the number of bits with value 1 in molecule B, and c is the number of bits with value 1 in both molecule A and B.

### DeepCarc Model Development

**FIGURE 1:**
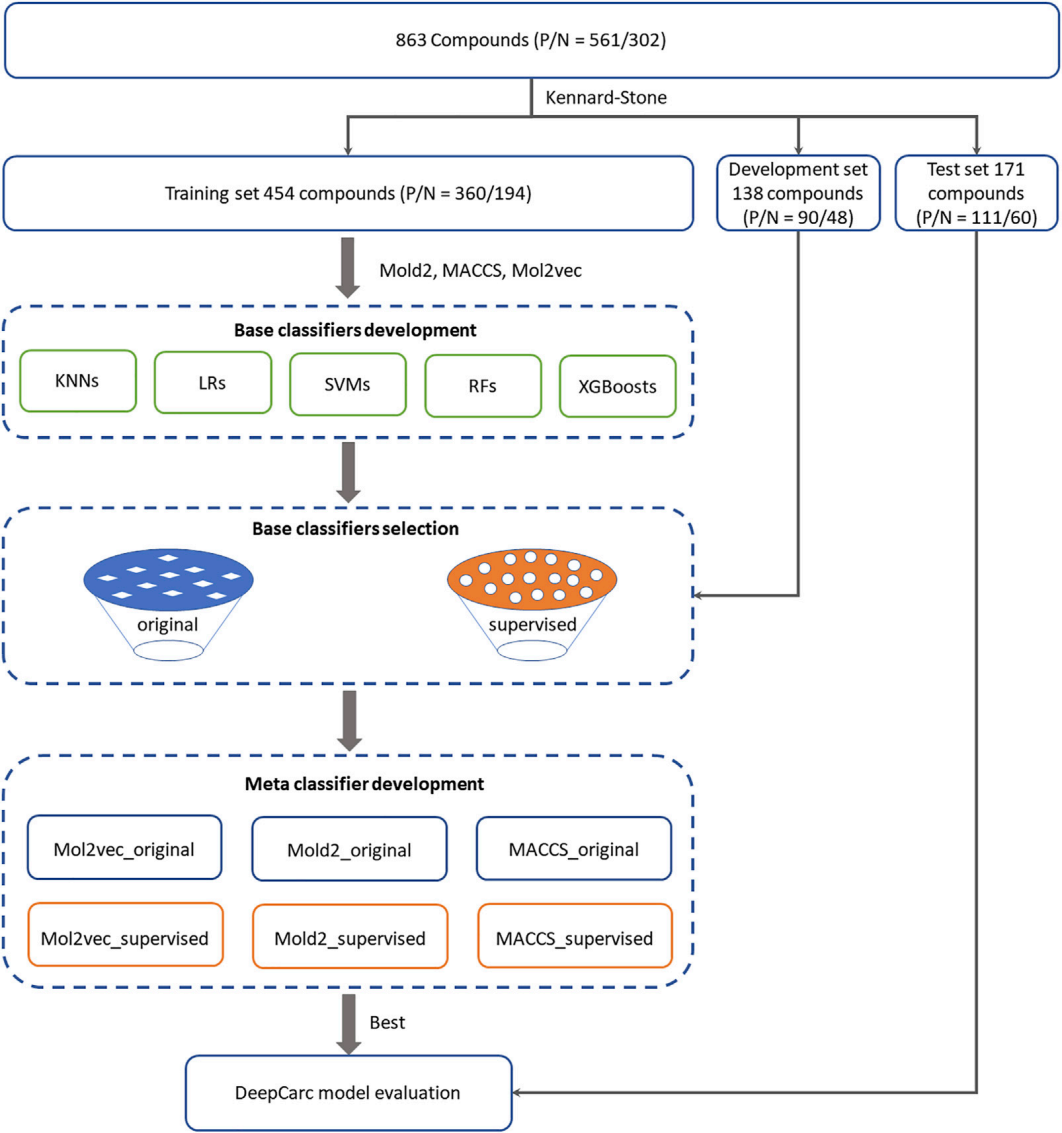
Overall workflow for the DeepCarc model including: (1) Data preparation. 863 compounds were split into training (554 compounds), development (138 compounds), and test (171 compounds) sets based on the Kennard-stone algorithm. (2) Base classifiers development. Five algorithms were used to develop the base classifiers from three different chemical representations, including Mol2vec, Mold2, and MACCS. Two base classifiers selection strategies were employed to select the optimized classifiers for meta classifier development. (3) Meta classifier development. With three chemical representations and two selection methods, six groups of base classifiers, including Mol2vec_supervised, Mol2vec_original, Mold2_supervised, were used Mold2_original, MACCS_supervised, and MACCS_original. The probability prediction from selected base classifiers was used to train the neural network. (4) Model evaluation. The DeepCarc model was evaluated on the independent test set.

DeepCarc model employed the same model architecture as DeepDILI (Li et al., 2021) by implementing a novel base classifier selection strategy (Figure 1). The input of NN is the probabilities output of the base classifiers (model-level representation). We hypothesized that no single learning algorithm could fit any modeling circumstance while different algorithms may provide complementary information. Therefore, the ensemble classifiers’ performance can improve to some extent.

### Base Classifier Development

Base classifiers were developed from five algorithms, including KNN, LR, SVM, RF, and XGBoost. The description of these five algorithms is as previously described (Cox, 1958; Cortes and Vapnik, 1995; Guo et al., 2003; Svetnik et al., 2003; Chen and Guestrin, 2016; Li et al., 2021). Comprehensive hyperparameter optimization was conducted for every algorithm using a bootstrap aggregating strategy (Breiman, 1996) (Supplementary Table S3). Specifically, 100 base classifiers were developed for each hyperparameter combination with randomly selected compounds from the training set (80%) and then validated on the development set. The best hyperparameter combination was obtained when the 100 base classifiers achieved the highest average Matthews correlation coefficient (MCC).

Two base classifier selection strategies were proposed, named original strategy and supervised strategy:

1) The original strategy was the base classifier selection approach used in the DeepDILI model. Specifically, 100 classifiers generated by each of the five algorithms with the best hyperparameters were rank-ordered based on MCC values. Only the ones with their MCC in the range of 5–95% percentile were chosen as optimized base classifiers for the meta-classifier development.
2) In the supervised strategy, we developed 1,000 base classifiers for each algorithm with the best hyperparameter combination from the training set. For each algorithm, the performance of every base classifier and the average performance of these 1,000 models was evaluated on both the training set and development set. Only the base classifiers with MCC values higher than the average MCC of both the training set and the development set were selected as the optimized base classifiers. Then, the optimized base classifiers selected from the five algorithms were combined for the meta-classifier development.

### Meta-Classifier Development

The meta-classifier NN aims to find the underlying relationship that transfers the optimized base classifiers’ information to target through linear or non-linear mathematical expression. In this study, a three-layer NN was developed as the meta-classifier for carcinogenicity prediction. Specifically, the input of NN came from the probabilities output of the optimized base classifiers (model-level representation) on the development set, which means a compound was represented by a vector of probabilities output from the optimized base classifiers. The hidden layer included 10 nodes with rectified linear unit (Relu) activation, stochastic gradient descent optimization, batch normalization, and a dropout of 0.5. The output layer used the sigmoid function to project the hidden layer information to probabilistic values of carcinogenicity prediction. The meta-classifier method was employed to develop six DeepCarc candidate models from the combination of three chemical representations (Mol2vec, Mold2, and MACCS) and two base classifiers selection strategies (original and supervised). For example, the candidate DeepCarc model of Mol2vec_original indicates the base classifiers were developed with the chemical representation of Mol2vec and filtered by the original base classifier selection method.

### DeepCarc Model Evaluation

The developed DeepCarc model performance was evaluated in the test set, including 171 compounds (111 carcinogens/60 non-carcinogens). The DeepCarc model was assessed by six performance metrics, including MCC, F1, accuracy, balanced accuracy (BA), sensitivity, and specificity, which were calculated using the following equations.

$$\mathrm{MCC}=\frac{\mathrm{TP}*\mathrm{TN}-\mathrm{FP}*\mathrm{FN}}{\sqrt{\left(\mathrm{TP}+\mathrm{FP}\right)*\left(\mathrm{TP}+\mathrm{FN}\right)*\left(\mathrm{TN}+\mathrm{FP}\right)*\left(\mathrm{TN}+\mathrm{FN}\right)}}$$ (3)

$$F1=\frac{2\mathrm{TP}}{2\mathrm{TP}+\mathrm{FP}+\mathrm{FN}}$$ (4)

$$\mathrm{accuracy}=\frac{\mathrm{TP}+\mathrm{TN}}{\mathrm{TP}+\mathrm{TN}+\mathrm{FN}+\mathrm{FP}}$$ (5)

$$\mathrm{BA}=\frac{\mathrm{sensitivity}+\mathrm{specificity}}{2}$$ (6)

$$\mathrm{sensitivity}=\frac{\mathrm{TP}}{\mathrm{TP}+\mathrm{FN}}$$ (7)

$$\mathrm{specificity}=\frac{\mathrm{TN}}{\mathrm{TN}+\mathrm{FP}}$$ (8)

The TP, TN, FP, and FN denote true positive, true negative, false positive, and false negative, respectively. In addition, the area under the receiver operating characteristic (ROC) curve (AUC) was also computed, where the ROC curve presents the performance of the classification model by measuring the relationship between true positive rate (TPR) against false positive rate (FPR) (Fawcett, 2006).

To investigate whether the probabilistic values yielded by DeepCarc could prioritize the compounds regarding carcinogenetic potential, we employed the Chi-Square test in different probabilistic thresholds (i.e., probabilistic value cut-off values were from 0.1 to 0.9 with a step of 0.1). Meanwhile, we calculated the positive predictive value (PPV) and negative predictive value (NPV) to investigate the discrimination power of probabilistic values for true positive and true negatives carcinogens, as shown in the following formulas:

$$\mathrm{PPV}=\frac{\mathrm{TP}}{\mathrm{TP}+\mathrm{FP}}$$ (9)

$$\mathrm{NPV}=\frac{\mathrm{TN}}{\mathrm{TN}+\mathrm{FN}}$$ (10)

### Comparative Analysis With Other Modeling Approaches

To further evaluate the proposed DeepCarc model, we compared DeepCarc with the optimized base classifiers developed from five algorithms, including KNN, LR, SVM, RF, and XGBoost. Furthermore, two ensemble methods, including the majority voting and average probability methods, were employed to justify the extra value of the proposed DeepCarc model over the conventional ensemble approaches. In the majority voting method, a consensus call of carcinogen/non-carcinogen was derived by the majority calls of the optimized base classifiers. In the average probability method, a new call was given to the non-carcinogen if the average probability of the optimized base classifiers was <0.5 and vice versa.

In addition, we compared the DeepCarc model against four other molecular-based DL models, including Text Convolutional neural network (CNN) from DeepChem (DC-TEXTCNN) (Wu et al., 2018), Chemistry Chainer-Neural Fingerprint (CH-NFP) (Duvenaud et al., 2015), Edge Attention-based Multi-relational Graph Convolutional Networks (EAGCNG) (Shang et al., 2018), and Convolutional Neural Network Fingerprint (CNF) (Tetko et al., 2019). The DC-TEXTCNN implemented the TEXTCNN based on chemical information, where the TEXTCNN was constructed to classify sentence tasks based on word representations. In the DC-TEXTCNN, the Simplified Molecular Input Line Entry System (SMILES) strings of molecules are the “sentence” input with the characters of the string represented as vectors. In the CH-NFP, the neural fingerprints are extracted from graphs of molecules and forwarded to a multilayer perceptron to make a classification prediction. The EAGCNG learns node features and attention weights in a graph convolutional network, where a molecular graph is represented by a real-valued attention matrix instead of a binary adjacency matrix. The CNF improves the molecule prediction by combining the synergy effect between CNN and the multiplicity of SMILES, which is used for feature extraction and data augmentation, respectively. These four DL models were developed from the Online Chemical Modeling Environment (OCHEM) website (https://ochem.eu/home/show.do). We used our training set and development set together to develop the models and then evaluated them on the independent test set.

### DeepCarc for Screening Carcinogenicity Potential of Compounds

The developed DeepCarc model was used as a screening tool for carcinogenicity risk detection in two external datasets, including DrugBank and Tox21. First, we collected 10,741 compounds from DrugBank database version 5.1.7 (Wishart et al., 2018), including approved and investigational drugs. After removing organometallics, heavy molecules, and the overlap compounds with our NCTRlcdb datasets, 9,814 investigated and approved drugs were kept (Supplementary Table S4). The output of predicted probabilistic values from the DeepCarc model was used to measure the carcinogenicity concern quantitatively. Second, we collected 8,410 compounds from the U.S. Tox21 program https://tripod.nih.gov/pub/tox21/, including food-additives, household cleaning products, medicines, and environmental hazard chemicals. The selection criteria of DrugBank were employed in the Tox21 dataset, and 7176 compounds were kept for screening by the DeepCarc model (Supplementary Table S5). We used the output of predicted probabilistic values from the DeepCarc model to quantitatively measure the carcinogenicity concern.

### Code Availability

All the models introduced above were developed with the open-source Python (version 3.6.5). The Mol2vec descriptors were generated from the source code https://github.com/samoturk/mol2vec. The open-source cheminformatics toolkit RDKit37 (version: 2020.09.1) was employed to construct the MACCS fingerprints. The Keras library version 2.0 with TensorFlow version 1.14 as the backend was used to develop NN classifiers. The scikit-learn package version 0.22 (Pedregosa et al., 2011) was applied to develop models with these four algorithms of KNN, LR, SVM, and RF. The open-source XGBoost library implemented on Python (version 3.6.5) was used to build all the XGBoost models. The scripts of all the models in this study are available at https://github.com/TingLi2016/DeepCarc.

## Results

### Discrimination Power of Chemical Representations

**FIGURE 2:**
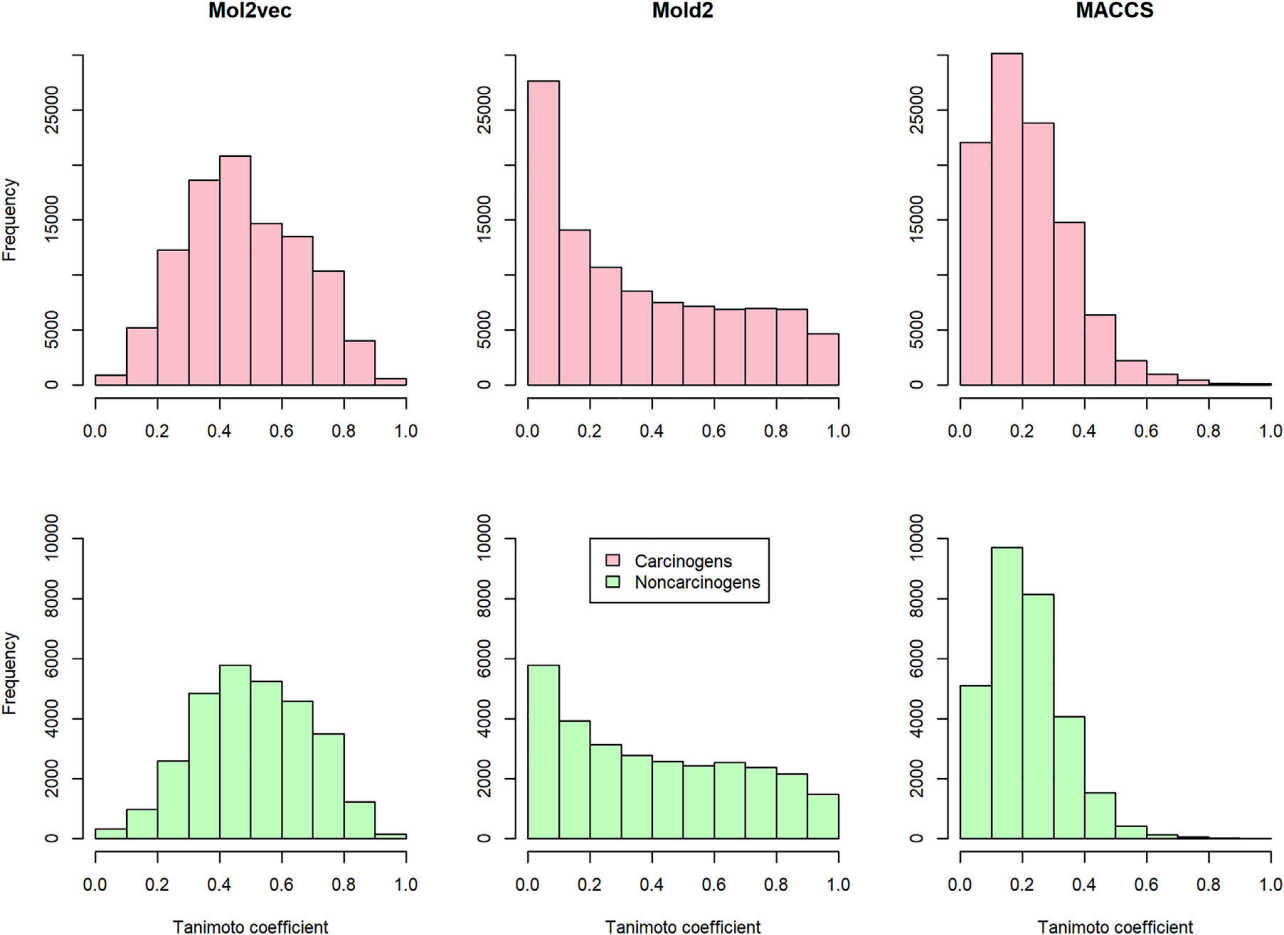
The distribution of the pairwise Tanimoto coefficients calculated from Mol2vec, Mold2, and MACCS: The pink and green indicate that the pairwise Tanimoto coefficients were calculated from the carcinogenic molecules and noncarcinogenic molecules, respectively.

To investigate the discrimination power of different chemical representations, we calculated the pairwise compound similarity (i.e., Tanimoto coefficients) among the compounds belonging to carcinogens (i.e., 450 compounds in training and development set) and non-carcinogens (i.e., 242 compounds in training and development set) with each chemical representation, respectively (Figure 2). Within each chemical representation (e.g., Mol2vec, Mold2, or MACCS), we observed a similar distribution of Tanimoto coefficients for carcinogens and non-carcinogens. For example, the average and standard deviation of Tanimoto coefficients were 0.479 ± 0.187 and 0.505 ± 0.182 for carcinogens and non-carcinogens based on Mol2vec chemical representation. Furthermore, the average and standard deviations of Tanimoto coefficients derived from Mold2 were 0.356 ± 0.297 and 0.401 ± 0.292 for carcinogens and non-carcinogens, whereas for MACCS they were 0.217 ± 0.143 and 0.214 ± 0.123. The Mol2vec tended to generate higher Tanimoto coefficients than Mold2 or MACCS, suggesting higher discrimination power of Mol2vec to cluster the compounds from the same category (i.e., carcinogens and non-carcinogens).

### Mol2vec With Supervised Selection Outperformed Other Combinations

**FIGURE 3:**
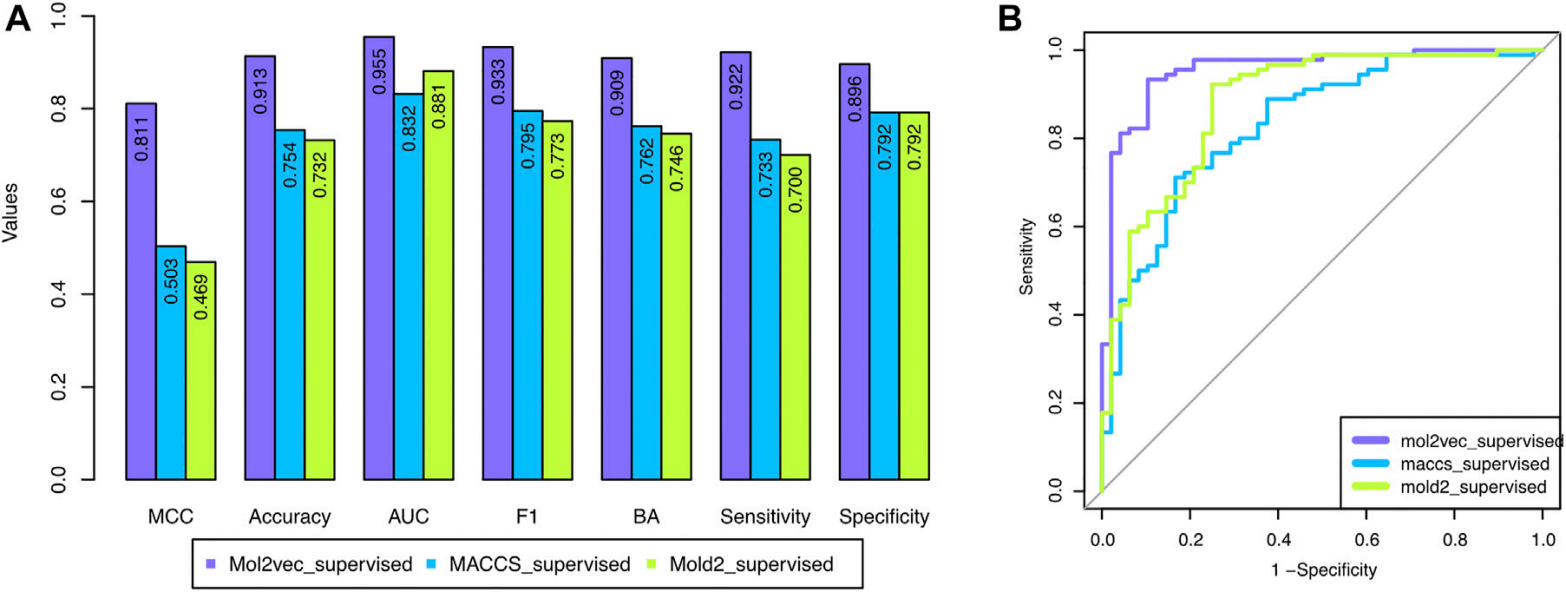
The performance of the developed DeepCarc models based on the proposed supervised base classifier selection strategy with the three chemical representations: the three chemical representations included Mol2vec, Mold2, and MACCS. **(A)**: Seven performance metrics; **(B)**: Area under the ROC curve.

To overcome the shortcoming of the base classifier selection strategy, we proposed a supervised classifier selection strategy by considering the performance from both training and development sets (see *Material and Methods*). Figure 3 depicted the development set performance using the proposed supervised base classifier selection strategy with the three chemical representations. The developed DeepCarc based on the Mol2vec with the proposed supervised base classifier selection strategy yielded the best performance across all the performance metrics (e.g., MCC = 0.811), which was much higher than that of Mold2 (i.e., MCC = 0.503) and MACCS (i.e., MCC = 0.469). Furthermore, the performance metrics of the DeepCarc model based on the proposed supervised base classifier selection strategy with Mol2vec were also much higher than those of the original strategy across all the performance metrics (Supplementary Figure S1). For example, the DeepCarc developed by the Mol2vec and supervised base classifier selection strategy had an improved rate of 18.57% compared to that of the original base classifier selection strategy (e.g., MCC = 0.684). Eventually, The DeepCarc model developed based on Mol2vec with the proposed supervised base classifier selection strategy consists of 296 RF, 285 LR, 277 KNN, 266 XGBoost, and 254 SVM which was considered as the optimized model for the following analysis.

### DeepCarc Effectively Augmented the Performance of Selected Base Classifiers

To evaluate whether the DeepCarc model could benefit from complementary information provided by different conventional machine learning algorithms, we compared the optimized DeepCarc model to the selected base classifiers developed from 5 ML algorithms (Table 1). For each machine learning algorithm, the average and standard deviation of the seven-performance metrics of the selected base classifiers were calculated for the development set and test set, respectively. The DeepCarc yielded the highest values in all the performance metrics except sensitivity (i.e., MCC = 0.811, accuracy = 0.913, AUC = 0.955, F1 score = 0.933, Balanced accuracy = 0.909, sensitivity = 0.922 and specificity = 0.896) compared to the selected base classifiers. For example, the DeepCarc made approximately an improvement of 77–127% of MCC over the selected base classifiers in the development set. Although the selected base classifiers achieved high sensitivities, they yielded very imbalanced performance regarding sensitivity (e.g., 0.991 ± 0.007 for RF) and specificity (0.212 ± 0.035 for RF). The performance followed the same trend in the test set, where the DeepCarc model achieved the highest value in MCC (0.432), accuracy (0.754), AUC (0.776), F1 (0.828), BA (0.688), and specificity (0.467). For instance, the DeepCarc made approximately 127–184% improvement in MCC over the selected base classifiers. Furthermore, the DeepCarc provided the most balanced performance regarding sensitivity (0.910) and specificity (0.467), whereas the selected base classifiers generated extremely lower specificity. In other words, the selected base classifiers tended to predict all the samples in the test set as carcinogens.

**TABLE 1:** The comparison between the base classifiers and DeepCarc performance on the development set and test set.

Data set	Model	MCC	Accuracy	AUC	F1	BA	Sensitivity	Specificity
Development set	DeepCarc	0.811	0.913	0.955	0.933	0.909	0.922	0.896
	XGBoost	0.458 ± 0.027	0.758 ± 0.011	0.785 ± 0.02	0.842 ± 0.006	0.659 ± 0.016	0.986 ± 0.007	0.331 ± 0.034
	LR	0.412 ± 0.024	0.746 ± 0.009	0.772 ± 0.012	0.830 ± 0.007	0.657 ± 0.016	0.95 ± 0.0260	0.364 ± 0.051
	SVM	0.408 ± 0.026	0.737 ± 0.010	0.754 ± 0.021	0.831 ± 0.005	0.626 ± 0.016	0.991 ± 0.012	0.261 ± 0.040
	KNN	0.372 ± 0.029	0.726 ± 0.009	0.694 ± 0.029	0.825 ± 0.005	0.612 ± 0.014	0.987 ± 0.010	0.236 ± 0.032
	RF	0.357 ± 0.032	0.720 ± 0.011	0.805 ± 0.018	0.822 ± 0.006	0.601 ± 0.016	0.991 ± 0.007	0.212 ± 0.035
Test set	DeepCarc	0.432	0.754	0.776	0.828	0.688	0.910	0.467
	XGBoost	0.187 ± 0.039	0.672 ± 0.007	0.715 ± 0.022	0.797 ± 0.004	0.536 ± 0.010	0.991 ± 0.003	0.081 ± 0.021
	LR	0.176 ± 0.033	0.670 ± 0.007	0.663 ± 0.017	0.794 ± 0.004	0.538 ± 0.011	0.981 ± 0.012	0.096 ± 0.028
	SVM	0.152 ± 0.039	0.665 ± 0.007	0.733 ± 0.020	0.793 ± 0.004	0.529 ± 0.009	0.986 ± 0.008	0.071 ± 0.020
	KNN	0.190 ± 0.037	0.672 ± 0.007	0.586 ± 0.031	0.797 ± 0.004	0.534 ± 0.009	0.993 ± 0.005	0.076 ± 0.019
	RF	0.163 ± 0.039	0.665 ± 0.006	0.700 ± 0.027	0.794 ± 0.003	0.524 ± 0.008	0.997 ± 0.004	0.051 ± 0.015

### DeepCarc Outperformed the State-of-the-Art Ensemble Classifiers

**FIGURE 4:**
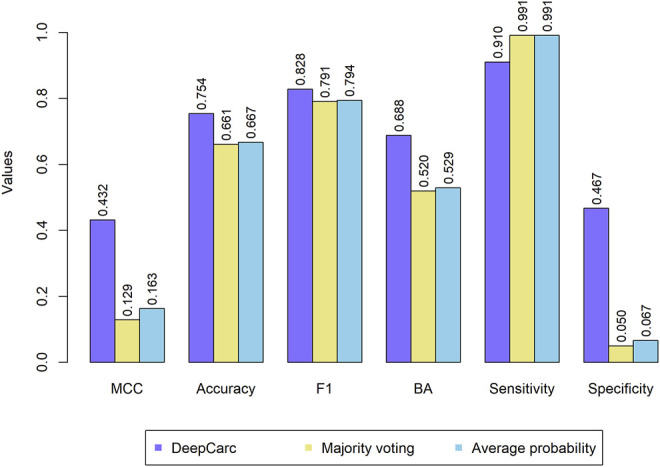
Ensemble models performance on the test set.

The comparison between DeepCarc and two state-of-the-art ensemble classifiers (i.e., majority voting and average probability) was also conducted on the test set (Figure 4). Consequently, the DeepCarc yielded better performance than the other two ensemble classifiers on MCC, accuracy, F1, BA, and specificity with an average improvement of 195.89, 13.55, 4.48, 31.17, and 698.29%, respectively. The majority voting and average probability generated the highest sensitivity (0.991 and 0.991, respectively), but with extremely low specificity (0.050 and 0.067, respectively), suggesting the proposed DeepCarc model could effectively optimize and combine the base classifiers.

### DeepCarc With Model-Level Representation Outperformed Molecule Representation-Based Deep Learning Models

To confirm the model-level representation and the molecule-based representation in carcinogenicity prediction, we compared the DeepCarc model with four other publicly available DL models, including DC-TEXTCNN, CH-NFP, EAGCNG, and CNF (Table 2). The model performance of these four DL models varied. Among these four deep learning models, DC-TEXTCNN resulted in the highest performance in the MCC of 0.392, accuracy of 0.735, F1 of 0.829, and sensitivity of 0.982. CH-NFP yielded the highest AUC of 0.776 and BA of 0.639, while EAGCNG achieved the highest specificity of 0.400. The imbalanced performance in sensitivity and specificity were also observed in these four deep learning models. DeepCarc outperformed these four deep learning models on MCC, accuracy, AUC, BA, and specificity. For example, DeepCarc improved 10–134% in MCC over the other four deep learning models.

**TABLE 2:** The model performance of DeepCarc and four advanced DNN models on the test set.

Models	MCC	Accuracy	AUC	F1	BA	Sensitivity	Specificity
DeepCarc	0.432	0.754	0.776	0.828	0.688	0.910	0.467
DC-TEXTCNN	0.392	0.735	0.719	0.829	0.627	0.982	0.271
CH-NFP	0.353	0.725	0.776	0.814	0.639	0.928	0.350
EAGCNG	0.328	0.713	0.682	0.800	0.641	0.883	0.400
CNF	0.185	0.673	0.636	0.796	0.541	0.982	0.100

### Predicted Probabilistic Values of the DeepCarc Model for Prioritizing Compounds on Their Carcinogenic Risk

To investigate the potential use of the DeepCarc model as the screening tool for prioritizing the carcinogenic risk, we employed the Chi-Square test to examine the correlation between carcinogen potential and predicted probabilistic values (Table 3). The *p* values yielded from the Chi-Square test were all less than 0.05 in probabilistic threshold from 0.2 to 0.9 with a step of 0.1, showing the strong correlation between the predicted probabilistic values of DeepCarc and the carcinogen risk. Furthermore, with the threshold increased, the PPVs increased from 0.663 to 0.887, meaning 88.7% compounds predicted with probabilistic values greater or equal to 0.9 were carcinogens. Meanwhile, the NPVs decreased as the threshold increased. The NPV yielded the highest value of 0.941 with the classification threshold value of 0.3 on the test set, indicating 94.1% of compounds predicted with a probabilistic value less than 0.3 were non-carcinogens. Altogether, the predicted probabilistic values of the DeepCarc model could be used as the indicators for prioritizing compounds regarding their potential carcinogenic risk.

**TABLE 3:** The relationship between predicted probabilistic values of DeepCarc and carcinogen risk.

Probabilistic threshold	DeepCarc prediction	Carcinogen		*p* Value	Positive predictive value	Negative predictive value
		Positive	Negative			
0.1	Predicted positive	110	56	5.188E-2	0.663	0.800
	Predicted negative	1	4			
0.2	Predicted positive	110	52	1.074E-3	0.679	0.889
	Predicted negative	1	8			
0.3	Predicted positive	110	44	1.51E-07	0.714	0.941
	Predicted negative	1	16			
0.4	Predicted positive	108	40	5.22E-08	0.730	0.870
	Predicted negative	3	20			
0.5	Predicted positive	101	32	4.22E-08	0.759	0.737
	Predicted negative	10	28			
0.6	Predicted positive	89	29	2.74E-05	0.754	0.585
	Predicted negative	22	31			
0.7	Predicted positive	81	22	7.18E-06	0.786	0.559
	Predicted negative	30	38			
0.8	Predicted positive	68	14	2.44E-06	0.829	0.517
	Predicted negative	43	46			
0.9	Predicted positive	47	6	9.85E-06	0.887	0.458
	Predicted negative	64	54			

### DeepCarc Is Employed to Screen DrugBank and Tox21 Compounds

**FIGURE 5:**
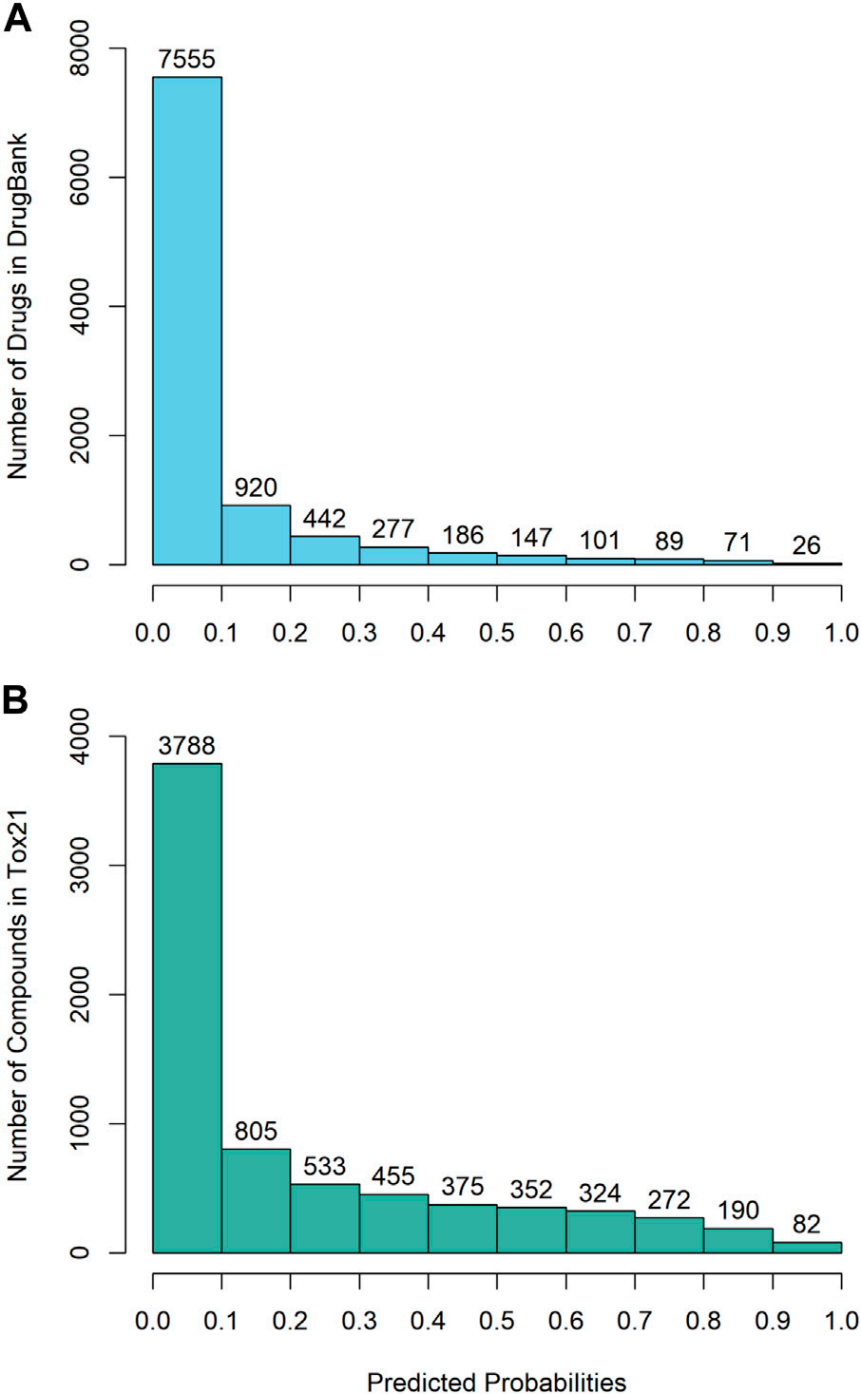
The probability distribution of the DeepCarc prediction of the compounds from **(A)** DrugBank; **(B)** Tox21.

The DeepCarc was used as a screening tool for identifying the carcinogenicity potential of the compounds from DrugBank (Figure 5A). The predicted probabilistic values ranging from 0 to 1 were split into 10 intervals with a size of 0.1. Of 9,814 compounds, there were 7,555 (i.e., 7555/9814 = 76.98%), 920 (9.37%), 442(4.50%), 277 (2.82%), 186 (1.90%) compounds with their predicted probabilities belong to the intervals of (0, 0.1), (0.1, 0.2), (0.2, 0.3), (0.3, 0.4), and (0.4, 0.5), respectively, indicating low carcinogenicity concern. In total, 434 compounds (4.42%) were predicted with probabilistic values ≥0.5, indicating compounds with carcinogenicity risk. Of 434 compounds, there were 26 compounds (0.26%) with the predicted probability ≥0.9, indicating high carcinogenicity concern. The predicted probabilistic value of each drug is included in (Supplementary Table S4).

The DeepCarc further screened the carcinogenicity potential of the compounds from the Tox21 (Figure 5B). Similarly, the predicted probabilistic values were separated into 10 intervals. Of the 7,176 compounds, there were 3,788 (i.e., 3788/7176 = 52.79%), 805 (11.22%), 533 (7.43%), 455 (6.34%), 375 (5.23%) compounds with their predicted probabilities belong to the intervals of (0, 0.1), (0.1, 0.2), (0.2, 0.3), (0.3, 0.4), and (0.4, 0.5), respectively, indicating low carcinogenicity concern. The other 1,220 (17.00%) compounds were predicted with probabilistic values ≥0.5, suggesting the compounds possessed carcinogenicity risk. There were 82 (1.14%) compounds with the predicted probabilistic value ≥0.9, suggesting high carcinogenicity concern (Supplementary Table S5).

## Discussion

Effectively evaluating the carcinogenicity of compounds is essential to improve the regulatory efficacy and promote public health. Performing a standard toxicity assay with two rodents (rats and mice) is expensive and time-consuming. Only a small proportion of compounds have been tested on carcinogenicity. Therefore, there is an urgent need for developing alternative methods to test carcinogenicity quickly and cost-effectively. A lot of computational models have been developed for prediction of carcinogenic potency. Some of these models can only be applied to specific chemical classes, and some were developed based only on rat’s carcinogenicity assay results. We developed a DeepCarc model to fill the gap by combining model-level representation generated from five conventional ML classifiers into a DL framework with Mol2vec descriptor and supervised base classifier selection strategy. The proposed DeepCarc model outperformed the optimized 5 ML classifiers, two state-of-the-art ensemble methods, and four molecule-based deep learning models. The developed DeepCarc model is publicly available through https://github.com/TingLi2016/DeepCarc.

The DeepCarc model was developed from the NCTRlcdb, which includes 863 compounds, and the carcinogenicity classification was built based on the carcinogenicity results of both rats and mice. The DeepCarc model was designed to predict the general carcinogens, which are non-organ specific. We investigated other reported machine learning-based prediction models with the NCTRlcdb data set (Liu et al., 2011; Tung, 2013; Tung, 2014; Beger et al., 2004). However, all the other reported prediction models aim to discriminate liver-specific carcinogens from others. Furthermore, samples used in these developed models varied from each other. One of the significant challenges of AI-based models towards real-world application is explainability. Here, we employed the Uniform Manifold Approximation and Projection (UMAP) to investigate the driving force of the proposed supervised base classifier selection strategy outperforming the original one (McInnes et al., 2018) (Supplementary Figure S2). The UMAP is a non-linear dimension reduction technique that captures the local relationships within the groups and the global relationships between different groups (Becht et al., 2019). We found that the supervised selection method had better discrimination power in distinguishing the carcinogens from non-carcinogens than the original selection method.

The DeepCarc model was compared with the other four DL carcinogenicity prediction models (DC-TEXTCNN, CH-NFP, EAGCNG, and CNF) using the chemical representation as a direct input. Different from the chemical descriptors used in the DeepCarc development, we explored three other different types of chemical representation, including SMILES strings (DC-TEXTCNN, and CNF), molecular graphs (CH-NFP), and molecular graphs with attention (EAGCNG). We also evaluated the impact on carcinogenicity prediction by enlarging the data set with the multiplicity of SMILES strings in the CNF model. DeepCarc outperformed these four DL models with the highest MCC of 0.432. The DC-TEXTCNN and CNF with SMILES strings as input had the highest sensitivity but lowest specificity. The CH-NFP and EAGCNG with the molecular graph as input reached higher specificity than the two DL models (DC-TEXTCNN and CNF) with SMILES string as input. Enlarging the data set by the multiplicity of SMILES string did not improve the performance in this carcinogenicity prediction.

Considering a large proportion of compounds in DrugBank and Tox21 without the carcinogenic test result, we employed the DeepCarc model to assess the carcinogenicity risk for the compounds from DrugBank and Tox21 to provide the information for further prioritizing the compounds for carcinogenicity assessment. We found that 1341 (1341/7176 = 18.69%) compounds were predicted with carcinogenicity risk in Tox21, which is much larger than 570 (570/9814 = 5.81%) drugs predicted with carcinogenicity risk in DrugBank. One of the possible reasons is that Tox21 includes environmental chemicals and household cleaning products, which are less likely to be evaluated by the carcinogenicity bioassay. However, there is a rigorous procedure to avoid carcinogens from getting marketed in drug development. A drug is required to take the 2-years carcinogenicity animal study if it will be used in treatment continuously for 6 months or more or with some special causes for concern, such as belonging to a class of the known carcinogens, showing evidence of precancerous changes in the chronic toxicity studies, and retaining in tissues for a long time (Rang and Hill, 2013). We conducted a literature survey to collect the compounds’ carcinogenic potential details with very high and low probabilities. However, we found little information on the carcinogenic testing results of these compounds. For example, Osimertinib was predicted with the carcinogenic probability of 0.928 and a study reported that it induced autophagy and apoptosis via reactive oxygen species generation in non-small cell lung cancer cells (Tang et al., 2017).

To investigate the potential artifact yield in the data split process, we randomly split the total 863 chemicals were into the different training set, development set, and test data set for 10 times to develop DeepCarc models. The low specificity of the test set compared to the development set is consistently observed in every newly developed DeepCarc model (Supplementary Figure S3). Identifying compounds with potential carcinogenic risks is very costly, time-consuming, and labor-intensive. A model with high sensitivity for detecting high carcinogenic risk compounds could be beneficial to narrow down a large number of compounds into a handled scale for further risk assessment. Considering the relatively low specificity and high sensitivity nature of the current DeepCarc model, we highly recommended positioning the model on screening of molecules in the early stage of development.

A low false-negative rate is one of the essential prerequisites to warrant the practical application of the prediction model in screening carcinogens. Therefore, we investigated the false-positives cases in our proposed DeepCarc model. There were 10 of 111 carcinogens predicted as non-carcinogens in the test set. The common structure analysis was employed for these 10 carcinogens. However, we did not find any common substructure, indicating only chemical information is insufficient to identify these carcinogens. Therefore, we recommend applying alternative approaches such as high-throughput *in vitro* toxicity assays (Li et al., 2017; Chiu et al., 2018) to further screen the non-carcinogens predicted by the DeepCarc to eliminate the false-negative cases in the real-world application.

The development of animal-free models is a new trend of modernized toxicity assessment. The 2-years bioassays in rats and mice are impossible to assess the carcinogenic potential of every compound efficiently and accurately. The DeepCarc model we developed could help prioritize potential carcinogens in the early stages of compounds development. Moreover, we hope our work will attract more interest to further exploring advanced artificial intelligence (AI) approaches for carcinogenic potency prediction.

## Data Availability

The original contributions presented in the study are included in the article/Supplementary Material, further inquiries can be directed to the corresponding authors.
