# Corrigendum: DeepCarc: Deep learning-powered carcinogenicity prediction using model-level representation

**DOI:** 10.3389/frai.2022.1046668

**Published:** 2022-11-28

**Authors:** Ting Li, Weida Tong, Ruth Roberts, Zhichao Liu, Shraddha Thakkar

**Affiliations:** ^1^Division of Bioinformatics and Biostatistics, National Center for Toxicological Research, US Food and Drug Administration, Jefferson, AR, United States; ^2^University of Arkansas at Little Rock and University of Arkansas for Medical Sciences Joint Bioinformatics Program, Little Rock, AR, United States; ^3^ApconiX Ltd., Alderley Edge, United Kingdom; ^4^Department of Biosciences, University of Birmingham, Birmingham, United Kingdom; ^5^Office of Translational Sciences, Center for Drug Evaluation and Research, US Food and Drug Administration, Silver Spring, MD, United States

**Keywords:** carcinogenicity, deep learning, QSAR, non-animal models, NCTRlcdb

In the published article, there was an error where the compounds were mismatched with the compound's prediction. A correction has been made to the Section “*DeepCarc Is Employed to Screen DrugBank and Tox21 Compounds*,” Paragraphs 1 and 2. The corrected section appears below:

“The DeepCarc was used as a screening tool for identifying the carcinogenicity potential of the compounds from DrugBank (Figure 5A). The predicted probabilistic values ranging from 0 to 1 were split into 10 intervals with a size of 0.1. Of 9,814 compounds, there were 7,555 (i.e., 7,555/9,814 = 76.98%), 920 (9.37%), 442 (4.50%), 277 (2.82%), 186 (1.90%) compounds with their predicted probabilities belong to the intervals of (0, 0.1), (0.1, 0.2), (0.2, 0.3), (0.3, 0.4), and (0.4, 0.5), respectively, indicating low carcinogenicity concern. In total, 434 compounds (4.42%) were predicted with probabilistic values ≥0.5, indicating compounds with carcinogenicity risk. Of 434 compounds, there were 26 compounds (0.26%) with the predicted probability ≥0.9, indicating high carcinogenicity concern. The predicted probabilistic value of each drug is included in **Supplementary Table 4**.

**Figure 5:**
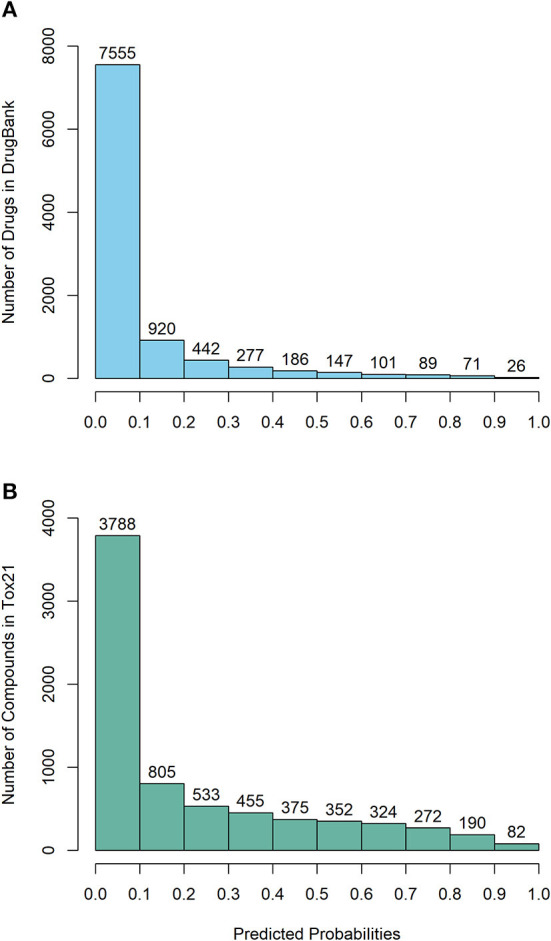
The probability distribution of the DeepCarc prediction of the compounds from **(A)** DrugBank; **(B)** Tox21.

The DeepCarc further screened the carcinogenicity potential of the compounds from the Tox21 (Figure 5B). Similarly, the predicted probabilistic values were separated into 10 intervals. Of the 7,176 compounds, there were 3,788 (i.e., 3,788/7,176 = 52.79%), 805 (11.22%), 533 (7.43%), 455 (6.34%), 375 (5.23%) compounds with their predicted probabilities belong to the intervals of (0, 0.1), (0.1, 0.2), (0.2, 0.3), (0.3, 0.4), and (0.4, 0.5), respectively, indicating low carcinogenicity concern. The other 1,220 (17.00%) compounds were predicted with probabilistic values ≥0.5, suggesting the compounds possessed carcinogenicity risk. There were 82 (1.14%) compounds with the predicted probabilistic value ≥0.9, suggesting high carcinogenicity concern (**Supplementary Table 5**).”

In the published article, there was also an error in Figure 5 as published. The probability distribution is incorrect due to a mismatched compounds' prediction. The corrected Figure 5 appears below.

In the published article, there was an error in **Supplementary Tables 4, 5** where the compounds were mismatched with the prediction. These files have now been updated in the original article.

The authors apologize for these errors and state that this does not change the scientific conclusions of the article in any way. The original article has been updated.

## Author disclaimer

The views presented in this article do not necessarily reflect current or future opinions or policies of the US Food and Drug Administration. Any mention of commercial products is for clarification and not intended as an endorsement.

## Publisher's note

All claims expressed in this article are solely those of the authors and do not necessarily represent those of their affiliated organizations, or those of the publisher, the editors and the reviewers. Any product that may be evaluated in this article, or claim that may be made by its manufacturer, is not guaranteed or endorsed by the publisher.

