# Supplementary figures and images for: DeepCarc: Deep Learning-Powered Carcinogenicity Prediction Using Model-Level Representation
Source: Front Artif Intell. 2021 Nov 18;4:757780. doi: 10.3389/frai.2021.757780 (PMC8636933; doi:10.3389/frai.2021.757780)

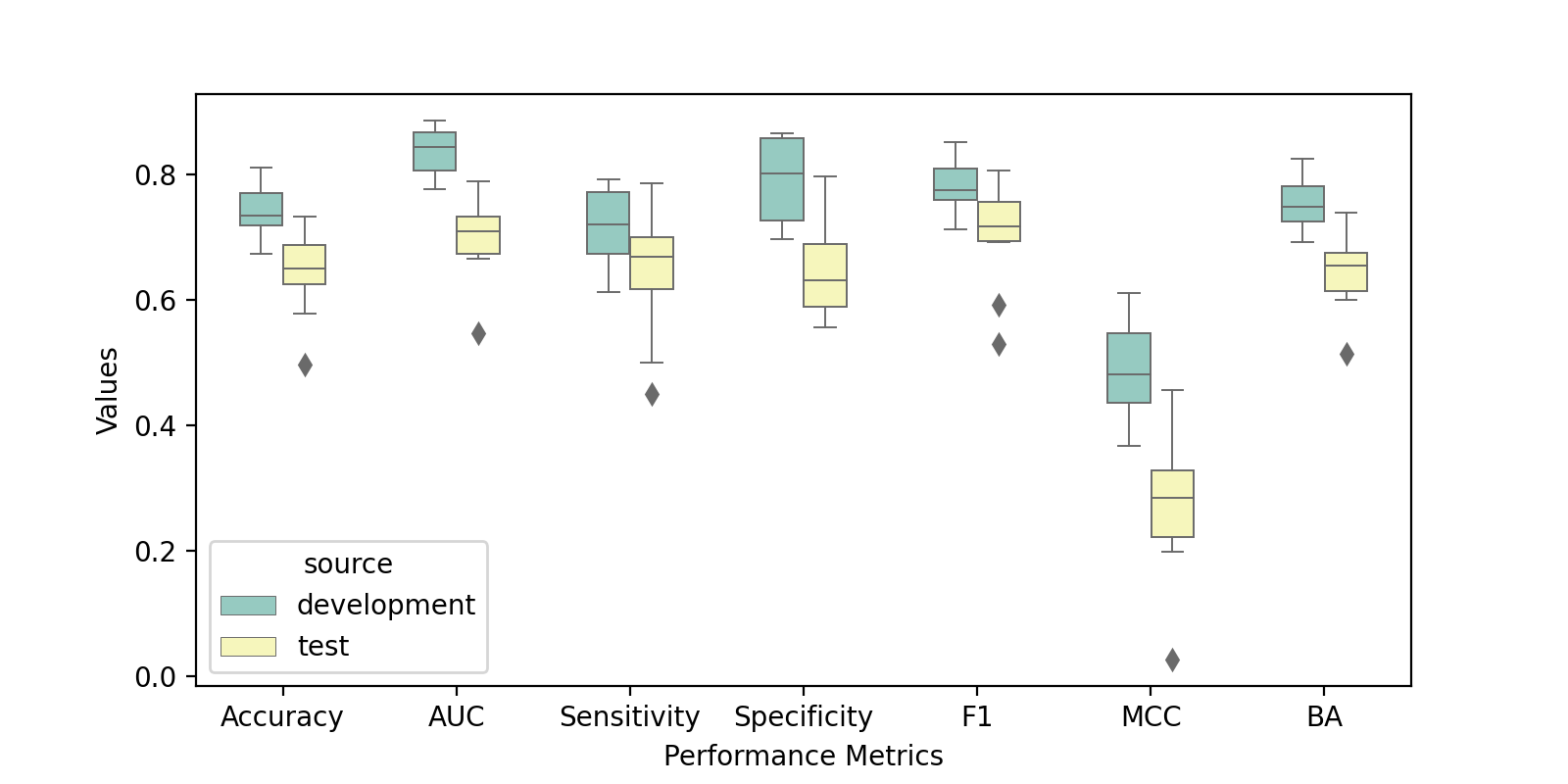

Supplement: Supplementary file 1 [file Image3.TIFF]

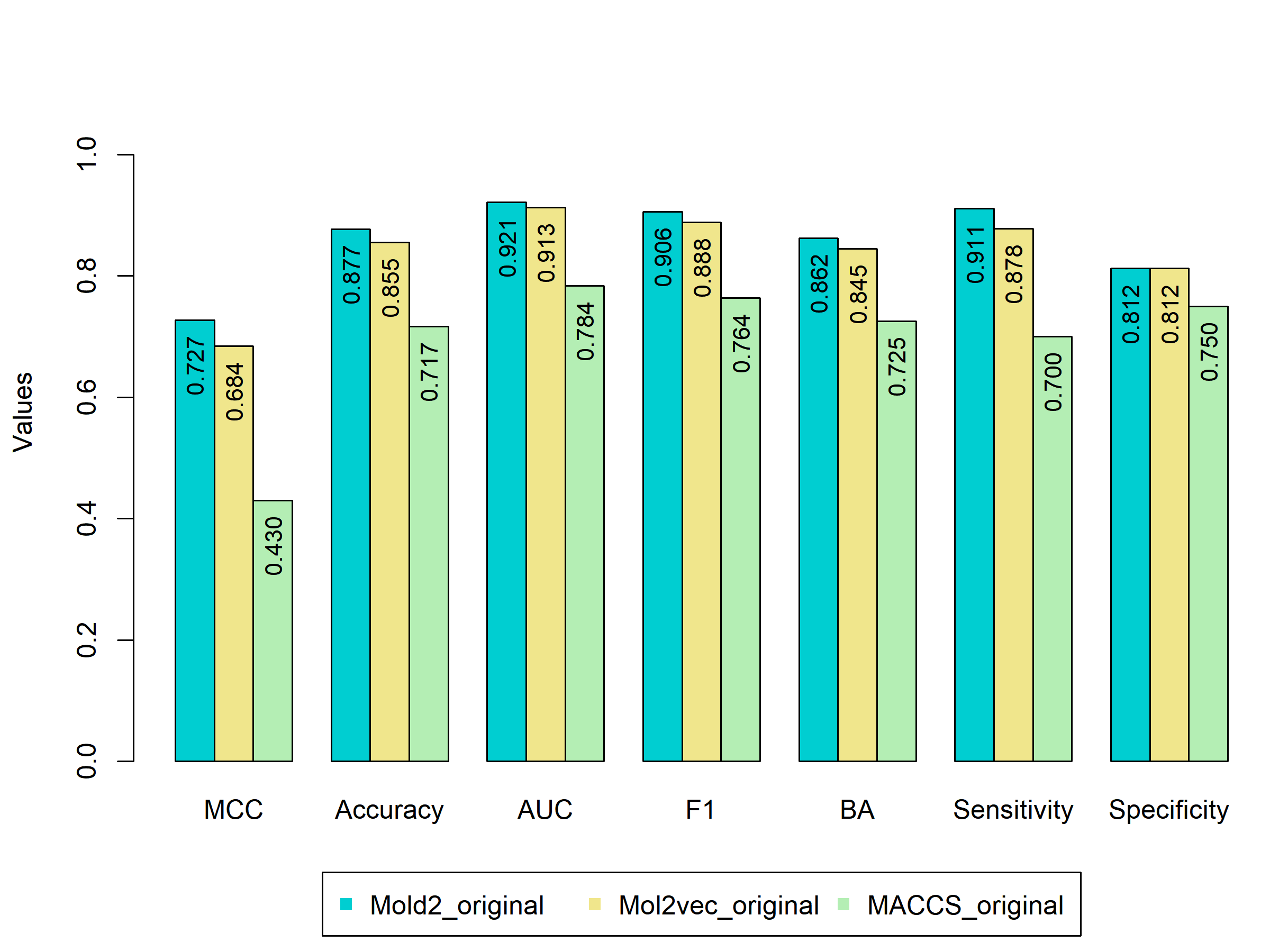

Supplement: Supplementary file 3 [file Image1.tiff]

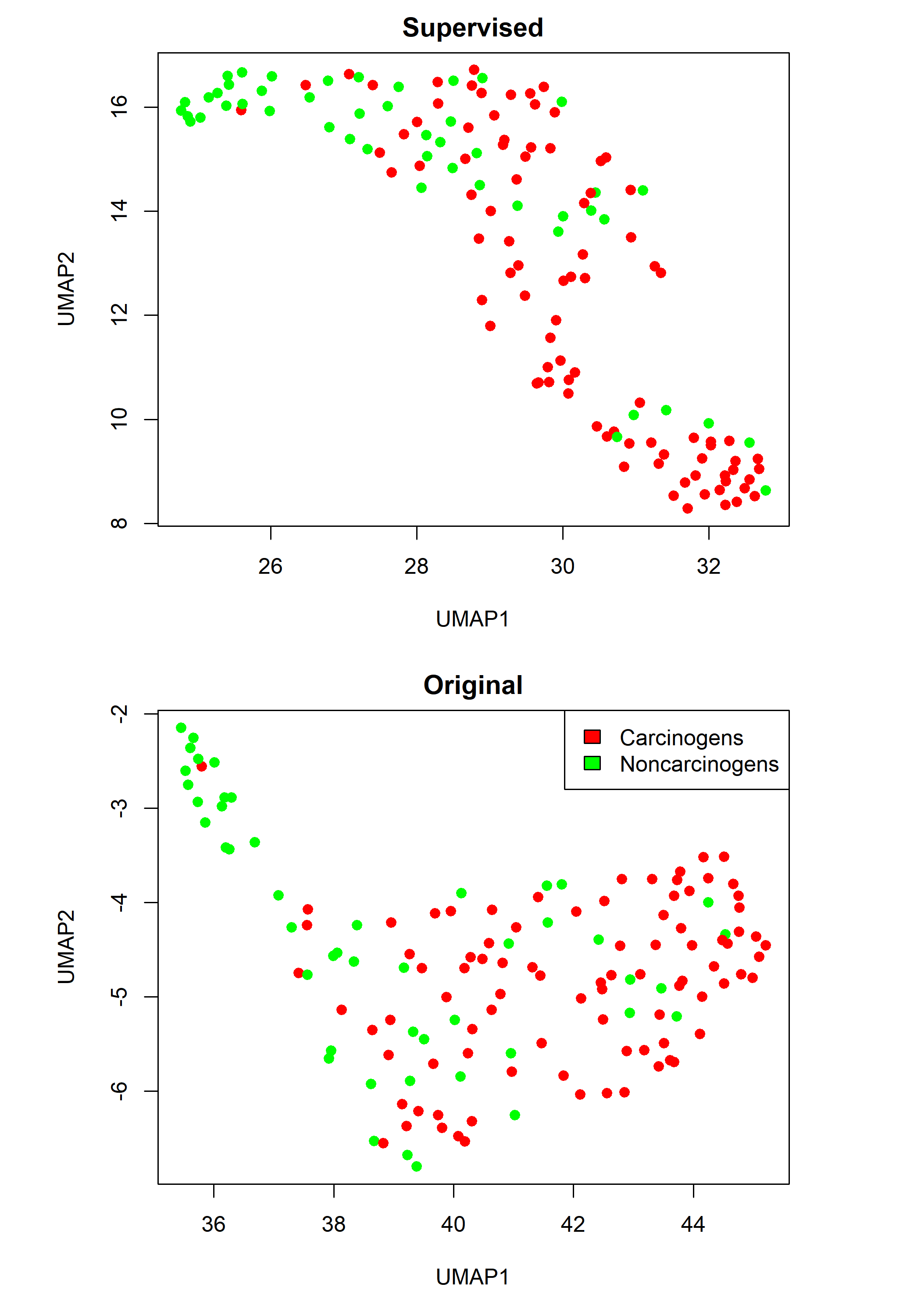

Supplement: Supplementary file 7 [file Image2.tiff]
